# Bone Marrow Mesenchymal Stem Cells Decrease the Expression of RANKL in Collagen-Induced Arthritis Rats via Reducing the Levels of IL-22

**DOI:** 10.1155/2019/8459281

**Published:** 2019-11-07

**Authors:** Fang Li, Xin Li, Guiyan Liu, Chong Gao, Xiaofeng Li

**Affiliations:** ^1^Department of Rheumatology, The Second Hospital of Shanxi Medical University, Taiyuan 030001, China; ^2^Endocrine Metabolism and Immune Center, Beijing Luhe Hospital, Capital Medical University, Beijing 101100, China; ^3^Department of Nephrology, Changzhi People's Hospital, Changzhi 046000, China; ^4^Department of Pathology, Joint Program in Transfusion Medicine, Brigham and Women's Hospital/Children's Hospital Boston, Harvard Medical School, Boston, MA, USA

## Abstract

**Objective:**

To investigate the transplantation effect of bone marrow mesenchymal stem cells (MSCs) on the expression of interlukin-22 (IL-22) and RANKL in collagen-induced arthritis (CIA) rats.

**Methods:**

32 CIA models were established. 16 CIA rats were transplanted with MSCs, and others were used as nontreatment CIA controls. The concentrations of IL-22 and RANKL in serum were detected by ELISA and those in synovial tissue of rats' joints by immunohistochemical staining. In addition, the expression of RANKL mRNA was measured by RT-PCR in the fibroblast-like synoviocytes (FLSs), cultured with IL-22 in vitro, which were delivered from the joints of CIA rats treated with or without MSCs.

**Results:**

The transplantation of MSCs into CIA rats relieved the destruction of joints, measured by AI score, X-ray, and histopathology. MSCs also reduced the expression of IL-22 and RANKL in serum by ELISA (*P* < 0.001) and similarly in FLSs by immunohistochemical staining. In vitro, IL-22 induced significantly the expression of RANKL mRNA in cultured FLSs in a dose-dependent manner, whereas this induction was significantly reduced in FLSs derived from CIA rats transplanted with MSCs (normal controls: *F* = 79.33, *P* < 0.001; CIA controls: *F* = 712.72, *P* < 0.001; and CIA-MSC rats: *F* = 139.04, *P* < 0.001).

**Conclusion:**

Our results suggest that the transplantation of MSCs can reduce the expression of RANKL in vivo by downregulating the levels of IL-22, thereby ameliorating the degree of RA bone destruction. This study provides a theoretical basis for a potential therapy of RA with MSCs, and IL-22 and RANKL may become two new targets to treat RA.

## 1. Introduction

Rheumatoid arthritis (RA) is a kind of chronic autoimmune diseases, characterized by cartilage and bone destruction [1]. Its pathogenesis remains uncertain. Although many drugs, including immunosuppressants, have been used conventionally to treat RA, the disease activity remission rate of RA is still very low [2, 3]. Furthermore, there is no method to repair the damaged cartilage and bone, so finding a new effective treatment of RA has been a hot topic for rheumatologists.

Osteoclasts, which are derived from a mononuclear phagocyte system, have been found to play a role in bone erosion and joint destruction. At present, a relative excess of bone resorption over bone formation has been considered a major cause of osteopenia and joint destruction in RA. Osteoclasts play an indispensable role in keeping balance between bone resorption and bone formation [4]. In recent years, many researchers have attempted to delay the progression of bone destruction in RA, by finding a method to inhibit or block the osteoclast differentiation.

RANKL is a key regulator factor of osteoclastogenesis and osteoclast differentiation [5]. Previous studies have shown that activated T cells can directly induce the preosteoclasts to osteoclasts by expressing the high levels of RANKL. Th1 and Th2 cytokines can inhibit osteoclast formation through interferon-*γ* (IFN-*γ*) and IL-4 [6]. Recently, fibroblast-like synoviocytes (FLSs) not only can express the high levels of RANKL the same as activated T cells but also can express TNF-*α*, IL-22, IL-1, IL-6, and prostaglandin E2 (PGE2), which stimulate the activation and differentiation of osteoclasts [7].

IL-22 was initially classified as a member of the IL-10 superfamily, but it has been placed within the smaller IL-20 subfamily recently [8]. It can promote the proliferation of FLSs and expression of monocyte chemoattractant protein (MCP). The incidence of arthritis and pannus went down in the CIA rats without IL-22. IL-22 might play a role in promoting inflammatory response and osteoclast formation in RA [9]. But whether IL-22 is associated with RANKL expression remains unclear.

Mesenchymal stem cells (MSCs) derived from marrow have a strong immune regulation effect [10]. In vitro, MSCs can inhibit the proliferation of T cells, the activation of NK cells, and the production of cytokines [11–13]. Interestingly, a previous study showed that MSCs cultured with high dose of RANKL and macrophage colony-stimulating factor (M-CSF) can inhibit the differentiation and activity of osteoclasts by secreting osteoprotegerin (OPG) [14] and RANKL can alter the function of MSCs to inhibit the formation of osteoclasts.

Other studies found that allogeneic MSC transplantation could improve the symptoms of arthritis and the pathology of synovium in CIA rats [15, 16]. However, whether transplanted MSCs affect the expression of IL-22 has been unknown. Hence, in this study, we transplanted MSCs to CIA rats to investigate the expression of IL-22 and RANKL in serum and synoviocytes. We assessed the effect of IL-22 on the expression of RANKL in FLSs in vitro. Our research results provided some theoretical basis for the treatment of RA with MSCs.

## 2. Materials and Method

### 2.1. Animals

Female Sprague-Dawley (SD) rats (160-180 g) were obtained from the Animal Research Center of the Shanxi Medical University and raised on the condition of 20°C room temperature and relative humidity (55 ± 10). Rats were able to move, eat, and drink freely.

### 2.2. Isolation and Characterization of MSCs

MSCs were isolated from SD rats (8–10 weeks). Briefly, the cells were flushed from two femurs and cultured in a T-175 flask under standard culture conditions of saturated humidity, pH 7.2, 5% CO_2_, and 37°C constant temperature. After forming the colonies, cells were flushed in complete expansion medium (CEM; Iscove's modified Eagle's medium, 9% horse and 9% fetal bovine serum, 1% penicillin and streptomycin, and 1% L-glutamine) at a density of 500 cells/cm^2^. MSCs were identified by cell surface markers (CD29, CD34, CD105, and CD45).

### 2.3. Preparation and Grouping of CIA Rats

Forty-eight SD rats were randomly divided into three groups: normal controls (*n* = 16), CIA controls (*n* = 16), and CIA-MSC group (*n* = 16), respectively. Approval from the local Institutional Animal Care and Use Committee was provided for all animal work. The rats of the healthy control group received the normal saline by vehicle injection. CIA models were established by immunizing SD rats with 0.1 ml mixed emulsion for two times (interval: two weeks), which consists of 400 *μ*g type II collagen (C6885-1g, Sigma, USA) and 0.1 ml Freund's complete adjuvant (FCA). The success of the model rats was judged by the X-ray of the left phalangeal joints and (or) the pathological changes of the joints under the light microscope. Rats in the CIA-MSC group were injected with 100 *μ*l PBS (1.0 × 10^7^ MSCs were resuspended in 1 ml PBS) through the tail vein. Normal and CIA controls were treated with saline of equal volume.

### 2.4. Clinical Evaluation

The clinical severity of arthritis was assessed weekly for each paw using the articular index (AI) score by two independent observers. The AI score was determined as follows: 0—no swelling or erythema, 1—slight swelling or erythema, 2—moderate swelling, 3—severe swelling, and 4—joint rigidity or malformation. Besides that, the degree of joint damage was evaluated by X-ray and histopathology of joints.

### 2.5. RANKL and IL-22 in Serum Were Measured by ELISA

The expression of serum RANKL and IL-22 was measured by ELISA (Bio-Swamp, Wuhan, China). A 96-well plate (Nunc) was coated with 40 *μ*l serum of rats and 10 *μ*l monoclonal antibodies against RANKL and IL-22 at 4°C.

### 2.6. Immunohistochemical Analysis of Ankle Joint FLSs

Immunohistochemical staining for IL-22 and RANKL was performed on sections of synovial tissue. Briefly, synovial tissue samples were obtained from rats of three groups. The tissue was fixed with 4% paraformaldehyde and embedded in paraffin. The 7 *μ*m thick sections were incubated at 4°C with a polyclonal anti-human RANKL antibody (Santa Cruz Biotechnology) and an anti-IL-22 antibody (R&D Systems). These samples were incubated with the secondary antibody, biotinylated anti-rabbit IgG, and streptavidin-peroxidase complex (Vector) for 1 hour, followed by incubation with 3,3-diaminobenzidine (Dako) for 5 minutes. The sections were counterstained with hematoxylin. The samples were photographed using an Olympus photomicroscope.

### 2.7. Extraction, Culture, and Treatment of FLSs

MSCs were transplanted to the CIA rats. About 1 month later, all rats were killed by neck removal. The rats were fixed on the back, and the skin was cut around the knee joints. We opened the ligament layer and removed the synovial tissue from the joint cavity. We rinsed it twice with DMEM containing a double antibody and cut it into tissue pieces of 1~2 mm^3^ size. The synovial tissue was added to the liquid, containing 0.4% type II collagenase for 2 hours. The unattached cells were transferred to 4 ml of 0.25% trypsin without EDTA. After being digested for 30 minutes, 8 ml of complete medium was added. Cells were centrifuged at 1500 rpm for 10 minutes, the supernatant was discarded, and the cells were resuspended. Then, we added complete medium and continued to culture. We observed the cell morphology under an inverted microscope at 24 h, 3 d, and 10 d of cell culture. To further identify FLSs, the third-generation synoviocytes were seeded on 24-well plates. After each well was full of cells, cells were flushed 2 times with PBS, fixed with formaldehyde for 10 minutes, incubated for 5 minutes, closed overnight by 5% BSA, added with an anti-vimentin antibody and anti-CD68 antibody separately for 1.5 hours, flushed 3 times with PBS, bound to goat anti-rat IgG for 40 minutes, flushed with PBS again, and incubated with SP for 20 minutes. After color development, counterstaining, and sealing, the expression of vimentin and CD68 was observed under a microscope. When we obtained FLSs, different concentrations of rrIL-22 (0, 0.1, 1, or 5 ng/ml) and FLSs were incubated for 1 week.

### 2.8. Measurement of RANKL mRNA Expression by RT-PCR

Total RNA was extracted from FLSs using RNAzolB (Biotex Laboratories), according to the manufacturer's instructions. RANKL mRNA expression was measured by reverse transcription polymerase chain reaction (RT-PCR) using the SuperScript reverse transcription system (Takara, Japan). The following primers were used in our study: RANKL, 5′-AGCCTTTCAAGGGGCCGTGC-3′ (forward) and 5′-GGGCCACATCGAGCCACGAA-3′ (reverse). The sequences of the housekeeping gene (*β*-actin) were as follows: 5′-CGGGAAATCGTGCGTGACAT-3′ (forward) and 5′-GAACTTTGGGGGATG CTCGC-3′ (reverse).

RT-PCR was performed to quantify the relative mRNA levels of RANKL using the SYBR® Premix Ex Taq™ RT-PCR kit (Takara), and the fluorescence curves were analyzed using LightCycler software (version 3.0, Roche Diagnostics), according to the manufacturer's instructions. The primers for RANKL were designed using the Primer 5.0 software. The process needed a 25 *μ*l amplification system. The reaction conditions are as follows: degeneration—1 cycle of 95°C for 30 sec; amplification—95°C for 5 sec, 40 cycles of 55-63°C for 20 sec, and 95°C for 60 sec; and melting curve analysis—30 sec (hold time) at 95°C, 15 sec at 71°C, and 30 sec (hold time) at 95°C. GAPDH is used as the housekeeping gene, and the quantity of mRNA was calculated by using the Ct value for amplification of RANKL. Relative gene expression was calculated by the 2^–ΔΔCt^ method.

### 2.9. Statistical Analysis

Statistical analysis was performed using SPSS 22.0 software. The normal distribution variables were presented as mean ± SEM, and the difference among the groups was analyzed by one-way analysis of variance (ANOVA). Comparison between two groups was analyzed by the Tukey method. The effect of IL-22 on RANKL was analyzed by the paired *t*-test. *P* < 0.05 was considered statistically significant. However, if the data were abnormal distribution, the variables were presented as interquartile ranges (P25, P75). The difference among the groups was analyzed by the rank sum test. *P* < 0.05 was considered statistically significant.

## 3. Results

### 3.1. Identification of MSCs

We successfully isolated and cultured MSCs, which were positive for CD29 and CD105, but negative for CD34 and CD45. Our results were consistent with previous reports [17, 18] (Supplemental Figure ([Supplementary-material supplementary-material-1])).

### 3.2. Therapeutic Effects of MSCs in CIA Rats

**Figure 1:**
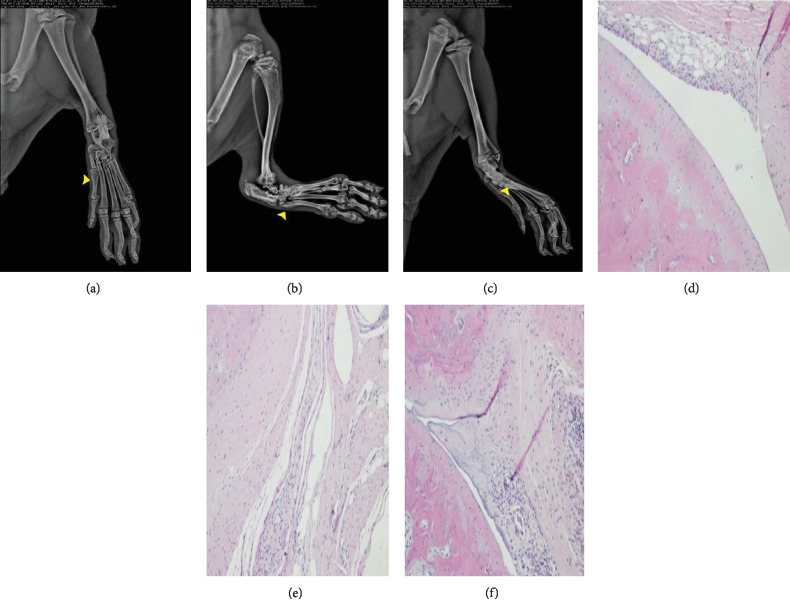
Therapeutic effects of MSCs in CIA rats. A decrease in severity of CIA following MSC transplantation is shown by X-ray (a, b, c) and HE stains (d, e, f). After injecting MSCs to CIA rats for 30 days, the X-ray showed that the joints of CIA rats (b, e) had osteoporosis and destruction compared with normal controls (a, d), and MSC transplantation reduced the damage of the joint with a wider joint space (c, f). MSCs: bone marrow mesenchymal stem cells; CIA: collagen-induced arthritis.

At day 30 after immunizing SD rats, X-ray of CIA rats showed that the joint space became narrow, even partly disappeared or deformed. Simultaneously, histopathology showed that the normal structure of joints was disappeared, the synovial membranes were thicker than the normal, and a large number of infiltrated lymphocytes were observed. Notably, 30 days after injecting MSCs to CIA rats, the X-ray showed that the joints had less osteoporosis and destruction and the joint space was less vague in the CIA-MSC group compared with CIA rats without treatment. Surprisingly, histology showed that the structure of the joint cavities was near normal, the synovial hyperplasia was not obvious, and the infiltration of inflammatory cells was less in the pathological section. The cartilage and bone of the CIA-MSC group were less damaged than those of the CIA control group (Figure 1). The AI score of CIA controls (7.02 ± 0.15) was higher than that of the CIA-MSC group (5.52 ± 0.18). The difference was statistically significant (*t* = 18.11, *P* < 0.0001).

### 3.3. The Expression of IL-22 and RANKL in Serum by ELISA

**Figure 2:**
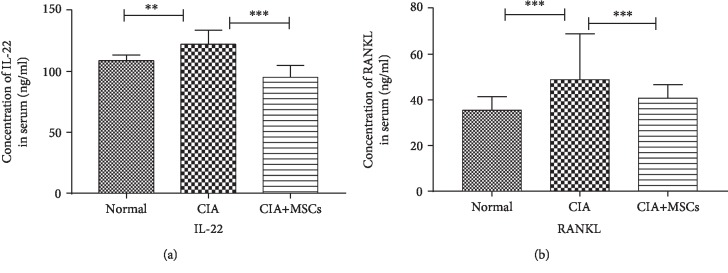
The expression of IL-22 and RANKL in serum by ELISA. The serum concentrations of IL-22 (a) and RANKL (b) in the three groups by ELISA. Normal controls (*n* = 16), CIA controls (*n* = 16), and CIA-MSC group (*n* = 16). When the rats were immunized with type II collagen, the expression of IL-22 and RANKL increased significantly (IL-22: *Z* = −2.752, *P* = 0.005; RANKL: *Z* = −4.373, *P* < 0.001). The highest levels of serum IL-22 and RANKL were found in CIA rats. In addition, using the MSCs to treat the CIA rats obviously reduced the levels of IL-22 and RANKL in serum (IL-22: *Z* = −4.109, *P* < 0.001; RANKL: *Z* = −3.411, *P* < 0.001). IL-22: interleukin-22; RANKL: receptor activator of nuclear factor-kappa B ligand. ^∗^Significant between-group difference (*P* < 0.05). ^∗∗^Significant between-group difference (*P* < 0.01). ^∗∗∗^Significant between-group difference (*P* < 0.001).

After the rats were immunized with type II collagen, the expression of IL-22 and RANKL increased significantly (IL-22: *Z* = −2.752, *P* = 0.005; RANKL: *Z* = −4.373, *P* < 0.001) compared with that of normal rats. The highest levels of serum IL-22 and RANKL were found in CIA rats among the three groups. Notably, transplantation of the MSCs to the CIA rats obviously reduced the levels of IL-22 and RANKL in serum (IL-22: *Z* = −4.109, *P* < 0.001; RANKL: *Z* = −3.411, *P* < 0.001) (Figure 2).

### 3.4. The Levels of IL-22 and RANKL in FLSs by Immunohistochemistry

**Figure 3:**
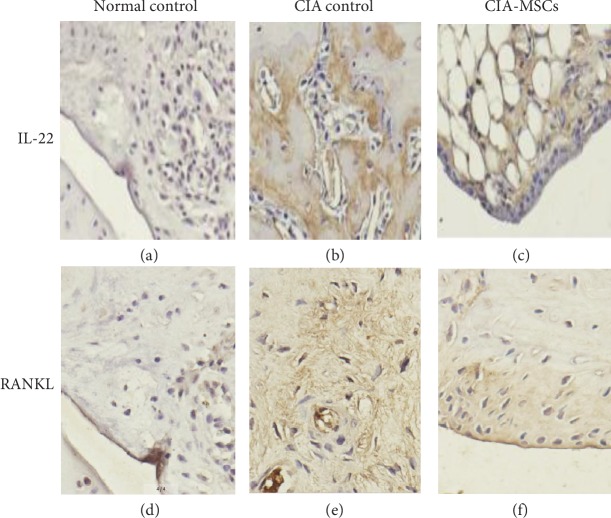
The differences of IL-22 and RANKL among the three groups in FLSs by immunohistochemistry. Representative images of immunohistochemical staining of IL-22 and RANKL in the joint tissue of normal controls (a, d), CIA rats (b, e), and CIA rats treated with MSCs (c, f) (*n* = 16). IL-22- and RANKL-expressing cells were brown in color (magnification: 100x). (a–c) IL-22 expression. (d–f) RANKL expression. When the rats were immunized with type II collagen, the expression of IL-22 and RANKL increased significantly. IL-22: interleukin-22; RANKL: receptor activator of nuclear factor-kappa B ligand; FLSs: fibroblast-like synoviocytes.

**Figure 4:**
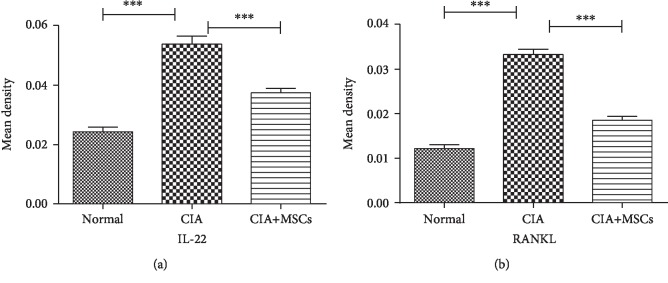
Density of IL-22 and RANKL in FLSs by immunohistochemistry. The levels of IL-22 and RANKL in the three groups were significantly different (IL-22: *F* = 86.546, *P* < 0.001; RANKL: *F* = 118.586, *P* < 0.001). The mean densities of IL-22 and RANKL in the CIA controls (0.0539 ± 0.0028 and 0.0333 ± 0.0021, respectively) were higher than those in the normal controls (0.0243 ± 0.0029 and 0.0121 ± 0.0015, respectively). After treating with MSCs, the mean densities of IL-22 and RANKL in FLSs were reduced (CIA-MSC group: 0.0375 ± 0.0026 and 0.0185 ± 0.0015, respectively). IL-22: interleukin-22; RANKL: receptor activator of nuclear factor-kappa B ligand; FLSs: fibroblast-like synoviocytes. ^∗^Significant between-group difference (*P* < 0.05). ^∗∗^Significant between-group difference (*P* < 0.01). ^∗∗∗^Significant between-group difference (*P* < 0.001).

The expressions of IL-22 and RANKL in FLSs by the immunohistochemical method were similar to those in serum by ELISA. They were significantly different among the three groups (IL-22: *F* = 86.546, *P* < 0.001; RANKL: *F* = 118.586, *P* < 0.001). The mean densities of IL-22 and RANKL (0.0539 ± 0.0028 and 0.0333 ± 0.0021, respectively) in FLSs of CIA controls were higher than those in the normal controls (0.0243 ± 0.0029 and 0.0121 ± 0.0015, respectively; *P* < 0.001, *P* < 0.001). After treating with MSCs, these mean densities were significantly decreased as compared with those in CIA rats without MSC transplantation (CIA-MSC group: 0.0375 ± 0.0026 and 0.0185 ± 0.0015, respectively; *P* = 0.001, *P* < 0.001) (Figures 3 and 4).

### 3.5. The Upregulation of RANKL mRNA in FLSs Stimulated with IL-22

**Figure 5:**
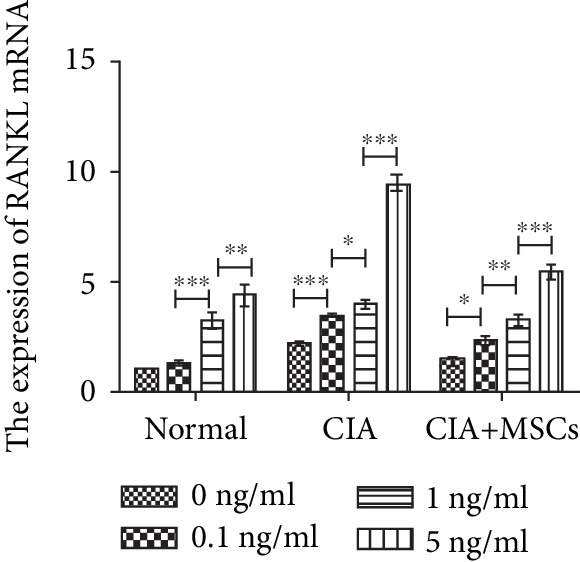
The expression of RANKL mRNA by RT-PCR in FLSs. The different concentrations (0 ng/ml, 0.1 ng/ml, 1 ng/ml, and 5 ng/ml) of IL-22 induced significantly the expression of RANKL mRNA in a dose-dependent manner. Compared between any two different dosing subgroups, the expression increases were significantly different (*P* < 0.05), except for 0 ng/ml and 0.1 ng/ml IL-22 in the normal group. Notably, after 5 ng/ml of IL-22 treatment, the levels of RANKL mRNA in CIA controls were higher than those in CIA rats transplanted with MSCs, indicating that MSCs downregulated the induction of IL-22 (normal controls: *F* = 79.33, *P* < 0.001; CIA controls: *F* = 712.72, *P* < 0.001; and CIA-MSC group: *F* = 139.04, *P* < 0.001). RANKL: receptor activator of nuclear factor-kappa B ligand. ^∗^Significant between-group difference (*P* < 0.05). ^∗∗^Significant between-group difference (*P* < 0.01). ^∗∗∗^Significant between-group difference (*P* < 0.001).

**Figure 6:**
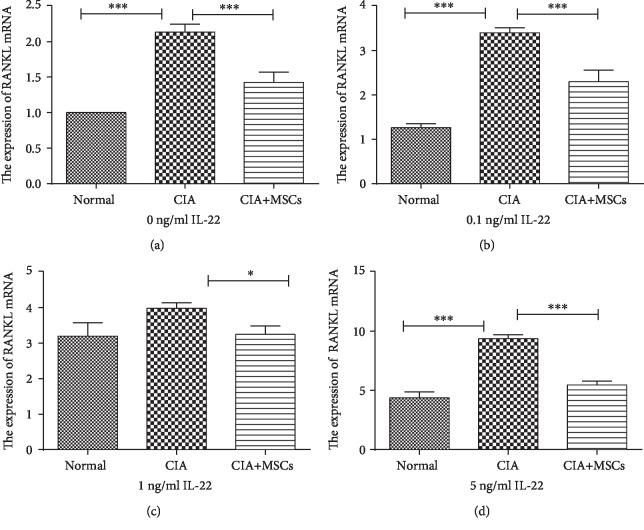
The expression of RANKL mRNA in FLSs treated with different doses of IL-22: (a) 0 ng/ml IL-22; (b) 0.1 ng/ml IL-22; (c) 1 ng/ml IL-22; (d) 5 ng/ml IL-22. The expression of RANKL mRNA in CIA controls was the highest among the three groups. The difference was statistically significant (0 ng/ml: *F* = 88.26, *P* < 0.001; 0.1 ng/ml: *F* = 116.83, *P* < 0.001; and 5 ng/ml: *F* = 129.37, *P* < 0.001), expect for adding 1 ng/ml IL-22 to the three groups (*H* = 5.422, *P* = 0.066). IL-22: interleukin-22; RANKL: receptor activator of nuclear factor-kappa B ligand; FLSs: fibroblast-like synoviocytes. ^∗^Significant between-group difference (*P* < 0.05). ^∗∗^Significant between-group difference (*P* < 0.01). ^∗∗∗^Significant between-group difference (*P* < 0.001).

After coculture of the different concentrations (0 ng/ml, 0.1 ng/ml, 1 ng/ml, and 5 ng/ml) of IL-22 and FLSs, the expression of RANKL mRNA gradually increased (normal controls: *F* = 79.33, *P* < 0.001; CIA controls: *F* = 712.72, *P* < 0.001; and CIA-MSC group: *F* = 139.04, *P* < 0.001). Even more, the levels of RANKL mRNA were dose-dependent. When we, respectively, added the same concentration of IL-22 to three groups, the expression of RANKL mRNA in CIA controls (0 ng/ml: *F* = 88.26, *P* < 0.001; 0.1 ng/ml: *F* = 116.83, *P* < 0.001; and 5 ng/ml: *F* = 129.37, *P* < 0.001) was the highest among them, except for 1 ng/ml IL-22 in the three groups (*H* = 5.422, *P* = 0.066) (Figures 5 and 6).

## 4. Discussion

RA is a kind of chronic inflammatory autoimmune disease, involving symmetrical joints, characterized by periarticular osteopenia and bone erosion [1]. Osteoclasts, the only cells in the body with the ability to dissolve bone tissue, are derived from hematopoietic stem cells. Excessive activation and proliferation of osteoclasts could increase bone resorption, promote bone destruction, and eventually lead to joint deformities [19]. Receptor activator of nuclear factor-*κ* B ligand (RANKL) is essential for the differentiation of osteoclasts. Hence, RANKL has been found to play a role in promoting the formation of osteoclasts in RA. Studies found that RA synovial fibroblasts can express the high levels of RANKL, like activated T cells. Besides the high expression of RANKL, RA synovial fibroblasts also can express many cytokines such as TNF-*α*, IL-1, IL-6, and PGE2, which can stimulate the activation and differentiation of osteoclasts.

IL-22 can be expressed by activated T cells, especially CD4^+^T cells. Thus, the increase in IL-22 is correlated with T cell-mediated diseases [20]. There is a large amount of IL-22 in synovial fluid of RA patients, which is associated with disease activity [21]. After IL-22-deficient rats were immunized with type II collagen, the incidence of arthritis and pannus decreased. It was speculated that IL-22 may play a role in promoting inflammatory response and osteoclast formation in RA [22]. Recently, studies showed that 1,25(OH)_2_D3 may inhibit osteoclastogenesis of RA-FLSs by downregulating RANKL expression. The process could be mediated by IL-22 [23]. IL-22, like other proinflammatory factors, has been shown to stimulate RA synovial fibroblasts and induce RANKL expression, thereby participating in the destruction of RA articular bone.

At present, two or more disease-modifying antirheumatic drugs (DMARDs) were used to treat RA in the early stage by helping preserve joints by blocking inflammation indirectly [24]. However, no drugs have been found to repair cartilage and bone damage until now. Therefore, looking for new and effective methods to treat RA has been a hot topic in the research of rheumatology.

MSCs are stem cells with high self-proliferation and multidirectional differentiation potential [10]. MSCs have a regulatory effect on both innate and adaptive immune responses [20, 25, 26]. MSCs can inhibit dendritic cell (DC) production and reduce the expression of costimulatory molecules CD80 and CD86 on human leukocyte DR antigen (HLA-DR) and antigen-presenting cells (APC). Under the action of MSCs, APC can reduce proinflammatory cytokine expression, such as IL-2, IFN-*γ*, and TNF-*α*, and increase IL-10 production, thereby inhibiting inflammation. In addition, MSCs can inhibit the proliferation and function of NK cells, and IFN-*γ* plays an important role in this process [27, 28]. Talking about the correlation between MSCs and adaptive immune response, studies found that MSCs can secrete immunosuppressive factors, chemokines, and adhesion molecules, which are important in effective T cell suppression, T cell proliferation, apoptosis, and differentiation [29]. In addition, B cells, as the second major cells, are related to adaptive immune responses. MSCs also inhibit B cell proliferation and activation. MSCs can release metalloproteinase-processed CC-chemokine ligand 2 (CCL2) to suppress the activity of STAT3 and reduce paired box 5 (PAX5), thereby reducing the production of immunoglobulins [30].

Now, transplantation of MSCs for the treatment of autoimmune diseases has become a research hotspot. Animal model studies found that MSC infusion can significantly improve autoimmune meningitis, multiple sclerosis, glomerulonephritis, systemic lupus erythematosus, and other autoimmune diseases [31]. So far, a few researches on MSCs were found in the treatment of RA model rats. They showed that the MSC transplantation, an effective treatment for RA, could reduce foot swelling and arthritis pathological scores in CIA rats [32–34].

Recently, many researchers have tried to explore the underlying molecular mechanisms of MSCs in the treatment of RA. In 2016, the results of Park et al. [35] demonstrated that MSCs could improve the clinical joint score and reduce joint inflammation and damage by significantly decreasing serum IL-1*β*, TNF-*α*, IL-6, and INF-*γ* and increasing IL-10, transforming growth factor-*β* (TGF-*β*), and regulatory T cell (Treg) levels. In addition, the data suggested that MSC transplantation may alleviate the severity of RA joints partially through suppressing miR-548e-mediated I*κ*B inhibition [36]. Also, some researchers believe that MSC transplantation could relieve the symptoms of arthritis by downregulating the expression of cartilage oligomeric matrix protein (COMP) on the synovial membrane and in the serum of CIA rats [37].

In recent years, researchers found that Th17 cells not only could secrete IL-17A and IL-17F but also produce cytokines, including TNF-*α*, IL-22, IL-26, and CCL20, and the chemokine receptor CCR6 on the cell surface [38]. MSCs have been found in vitro to prevent the differentiation of immature CD4+T cells into Th17 cells. Besides that, they could reduce the activation of Th17 cells, IL-17, IL-22, INF-*γ*, and TNF-*α*. In the preliminary researches, we have found that allogeneic MSC transplantation into CIA rats could ameliorate the pathological outcome of synovial arthritis through upregulating regulatory T (Treg) cells, promoting the expression of Foxp3 mRNA, secreting TGF-*β*, inhibiting the proliferation of B lymphocytes, and reducing contents of TNF-*α* and IL-17. However, it is not clear whether MSCs could reduce the expression of RANKL by relieving the levels of IL-22 in CIA rats. Our studies highlighted the relationship between IL-22 and RANKL in the CIA rat models treated with MSCs.

In our study, MSC transplantation reduced the infiltration of inflammatory cells around the joint, the destruction of cartilage and bone, and the AI score. The results were similar to the outcome of previous studies. Studies showed that activated T cells could promote the expression of IL-22 and RANKL. Then, MSCs could inhibit the expression of T cells. We assumed that the MSCs alleviated the symptoms of RA mainly by reducing the levels of IL-22 and RANKL. By the ELISA and immunohistochemistry, the levels of IL-22 and RANKL were much lower in the CIA-MSC group than in CIA controls. Those results are consistent with our hypothesis.

Interestingly, under the action of IL-22, MSCs can exert immunosuppressive effects in vitro. Studies found that under the specific induction conditions in vitro, MSCs have the potential to differentiate into various tissue cells, such as fat, bone, cartilage, and muscle. MSCs can be used to repair tissue damage caused by diseases. By paracrine signaling, MSCs can secrete not only a variety of biologically active molecules, including growth factors, cytokines, and hormones, but also extracellular vesicles (EVs) [39]. EVs can be used as a carrier to transport a variety of signal substances between cells, which play an important role in the intercellular communication. According to different intracellular origins, EVs are divided into two types, microvesicles and exosomes. Microvesicles, diameters between 100 nm and 1 *μ*m, are released by cell membrane sprouting. Exosomes are produced by polycystic bodies, released by fusion with the plasma membrane. In the past years, MSC-derived EVs have become a potential treatment for immune-related diseases, and MSCs could exert some of their biological properties through secreted EVs. Studies have found that MSCs-EVs can decrease the secretion of proinflammatory cytokines (IL-1*β*, IL-2, IL-6, IFN-*γ*, TNF-*α*, and IL-17A) and increase the production of anti-inflammatory cytokine TGF-*β* [40]. In our study, IL-22 could increase significantly the expression of RANKL mRNA in a dose-dependent manner in vitro. At the same time, MSCs could decrease the expression of RANKL under the stimulation with the same dose of IL-22 in vitro. We guess that MSCs may downregulate the expression of IL-22 by secreting EVs, thereby reducing the expression of RANKL. However, the detailed mechanism is still unclear. We need to verify our conjecture in our future experiments.

## 5. Conclusion

Our results demonstrated that MSCs could ameliorate the degree of RA bone destruction by reducing the expression of IL-22-mediated RANKL. This study may provide a theoretical basis for a potential therapy of RA with MSCs. IL-22 and RANKL may become the new targets to treat RA.

## Data Availability

The data used to support the findings of this study are available from the corresponding author upon request.

## Supplementary Materials

Supplementary Materials Histograms showed levels of surface antigen expression and their corresponding isotype controls. On the surface of MSCs, the expression of CD29 and CD105 was positive and the expression of CD34 and CD45 was negative.
