# Supplementary material for: Bone Marrow Mesenchymal Stem Cells Decrease the Expression of RANKL in Collagen-Induced Arthritis Rats via Reducing the Levels of IL-22
Source: J Immunol Res. 2019 Nov 7;2019:8459281. doi: 10.1155/2019/8459281 (PMC6885301; doi:10.1155/2019/8459281)
Supplement: Supplementary Materials — Histograms showed levels of surface antigen expression and their corresponding isotype controls. On the surface of MSCs, the expression of CD29 and CD105 was positive and the expression of CD34 and CD45 was negative. [file 8459281.f1.pdf]

Supplemental Figure: Identification of MSCs' phenotype by flow cytometry.

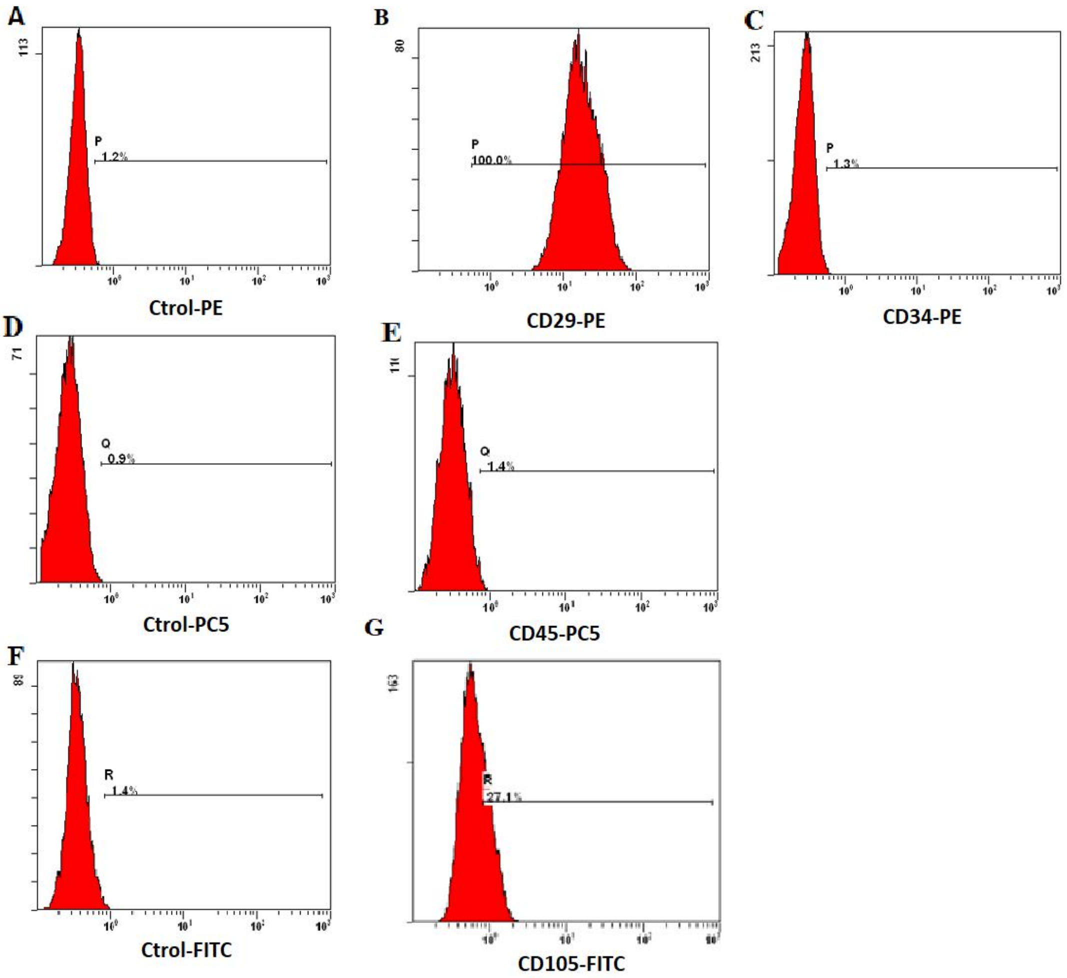

Histograms showed levels of surface antigen expression and their corresponding isotype controls. On the surface of MSCs, the expression of CD29, CD105 were positive, the expression of CD34, CD45 were negative.

A: isotype-PE control; B: MSCs marker CD29-PE (positive); C: CD34-PE; D: Isotype-PE-CY5 control; E:CD45-PE-CY5; F:Isotype-FITC-control; G: MSC-CD105 (positive).

Bone marrow mesenchymal stem cells=MSCs
